# MagnetoShield: A Novel Open-Source Magnetic Levitation Benchmark Device for Mechatronics Education and Research

**DOI:** 10.3390/s24020538

**Published:** 2024-01-15

**Authors:** Gergely Takács, Jakub Mihalík, Martin Gulan, Anna Vargová, Erik Mikuláš, Štepán Ožana

**Affiliations:** 1 AutomationShield.com Open-Source Initiative, 812 31 Bratislava, Slovakia; gergely.takacs@gmail.com (G.T.); jakub.mihalik33@gmail.com (J.M.); anna.vargova@stuba.sk (A.V.); mikulaserik@me.com (E.M.); 2 Institute of Automation, Informatization, and Measurement, Faculty of Mechanical Engineering, Slovak University of Technology in Bratislava, 812 31 Bratislava, Slovakia; 3 Department of Cybernetics and Biomedical Engineering, Faculty of Electrical Engineering and Computer Science, VSB—Technical University of Ostrava, 17. listopadu 2172/15, 708 00 Ostrava, Czech Republic; stepan.ozana@vsb.cz

**Keywords:** magnetic levitation, control education, mechatronics, open hardware, Arduino

## Abstract

This article presents an open-source device illustrating the well-known magnetic levitation experiment. The uniqueness of this particular device lies in its exceptionally small dimensions, affordability and availability, which makes it a perfect design for take-home experiments for education but it can also serve as a referential design for testing various control algorithms in research. In addition, this paper provides a comprehensive hardware design for reproducibility along with the detailed derivation of the mathematical model, system identification and validation. Moreover, the introduced hardware comes with an easy-to-use open-source application programming interface in C/C++ for the Arduino IDE, Simulink and CircuitPython. REXYGEN, another environment similar to Simulink, had also been used to demonstrate the capabilities of the MagnetoShield.

## 1. Introduction

Over the past few decades, the fields of industry and technology have seen significant changes due to the ever-increasing power of computation and the decreasing cost of communication. These changes have had a profound impact on various industries, including engineering. As a result, mechatronics, which is an interdisciplinary branch of engineering, is also evolving. The changes in industry and technology have led to modifications in the requirements for engineers, and they are now often expected to take on the role of system integrator. This involves connecting the various components of a complex system or product. To carry this out successfully, engineers must have a deep understanding of control frameworks and tools, as well as a broad technical knowledge across various fields. Therefore, the changes in industry and technology have led to the transformation of mechatronics and the evolution of the role of the engineer [1].

Overall, the changing requirements for engineers have had a significant impact on the curriculum and delivery of engineering courses too. Educational institutions are adapting to ensure that their students are equipped with the necessary skills and knowledge to succeed. Simultaneously, nowadays, students are typically characterized as being more diverse, tech-savvy, and demanding in terms of the support and resources they require. They expect to be able to use technology as a tool for learning and to have access to resources and information online [2]. They also tend to desire more personalized and experiential learning experiences, with opportunities to work independently and in groups [3]. Furthermore, the recent COVID-19 pandemic has also accelerated the adoption of online and remote learning, and personalized approach [2].

As technology continues to advance, students are increasingly able to access laboratory devices and simulations remotely, allowing for greater flexibility and convenience. However, there is still value in traditional, hands-on experimentation. To address this, new solutions that are both portable and affordable are emerging, such as miniaturized lab kits that can be taken home or used in small groups. These types of experiments can help students to develop problem-solving and analytical skills while still maintaining the advantage of studying independently. In fact, these low-cost lab kits can be seen as complementary to virtual and remote access laboratories, as they provide students with the opportunity to encounter “real” problems, such as measurement noise, integration, and system design, all while studying individually, or even at home.

Pocket-sized and take-home laboratory kits frequently take their inspiration from the larger, dedicated laboratory systems commonly found in university settings. As a result, they often incorporate many of the same types of academic benchmarks. While academic benchmarks such as inverted pendulums, motor speed control systems, and temperature control experiments have long been used in engineering education, they may not always have a direct application in industry. Rather, they are often used as testbeds for developing and testing control algorithms and other theoretical concepts. In contrast, magnetic levitation (maglev) systems have a more direct application in industry and technology, particularly in the field of mechatronics. For example, maglev technology is used in high-speed trains for efficient and fast transportation, as well as in precision positioning systems and frictionless bearings. In addition, magnetic levitation allows for simulating microgravity conditions to advance bottom-up tissue engineering and regenerative medicine for space exploration [4], or for testing various control algorithms [5].

**Figure 1:**
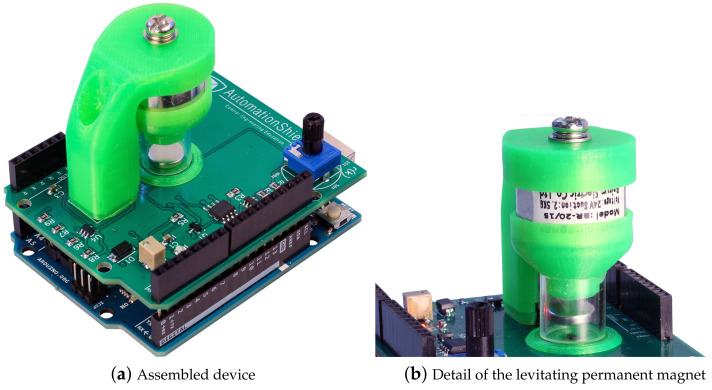
Photograph of the MagnetoShield.

Magnetic levitation systems have also been used in engineering education and research for various purposes. For example, they have been utilized to demonstrate nonlinear system modeling and advanced control techniques [6], as well as control based on neural networks [7,8] and fuzzy logic [9]. Additionally, the maglev system has been integrated into networks of remote laboratories [10] and used in applications such as data acquisition and processing [11]. In this paper, we present a similar compact, affordable take home-laboratory—the MagnetoShield; depicted in Figure 1.

In contrast to existing commercial maglev devices with high costs reaching several thousand dollars (as seen in studies like [6,8,10]), our presented solution, the MagnetoShield, stands out as a low-cost alternative. Unlike cheaper custom-made options in [7,9], which lack comprehensive hardware documentation and standardized features, the MagnetoShield provides an affordable yet standardized platform. Notably, it is an open-source project with readily available documentation, enabling anyone to construct a similar device. This accessibility facilitates the replication of experiments showcased, as well as the creation of personalized solutions. Moreover, the MagnetoShield’s compatibility with Arduino microcontroller boards and similar devices empowers users to tailor experimental configurations, fostering exploration into various aspects of mechatronics and related disciplines, thereby serving as an invaluable tool for education and research in control theory.

MagnetoShield can be used for hands-on experiments in control systems and mechatronics education. It is built as an Arduino expansion module, making it compatible with a wide range of microcontroller prototyping boards. The open-source hardware design uses off-the-shelf components and 3D printing technology, resulting in a minimal material cost. It also has an open-source application programming interface and can be used for teaching modeling, system identification, closed-loop control, or even conducting research on a budget. The device was developed as part of the larger initiative AutomationShield, which aims to create open-source low-cost educational tools for control systems and mechatronics [12,13]. We have to mention that there is other similar hardware documented, e.g., Ref. [14] proposes a compact, low-cost, single-board electromechanical system for teaching modeling, dynamics, and control of mechatronic systems. The system has actuators, sensors, and power electronics implemented on a 70 × 100 mm printed circuit board. Another interesting use of low-cost maglev hardware is documented in a case study [15], where students obtain inadequate hardware, and the task is to implement improvements by creating their own desktop-sized systems with analog control. The uniqueness of our hardware lies not only in its small dimensions and affordability, but also in its reproducibility thanks to the open documentation [12] and the support within the ecosystem of the AutomationShield project.

**Figure 2:**
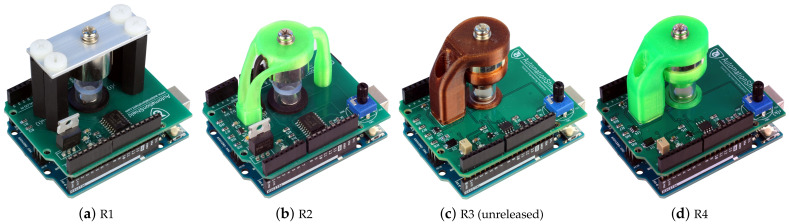
Evolution of the MagnetoShield hardware.

The current paper builds upon the hardware prototype of the device (see release R2 in Figure 2) introduced in our earlier work [16], and presents an upgraded version of the MagnetoShield, aimed specifically at enhancing the teaching of automatic control and mechatronics at both undergraduate and graduate levels.

In terms of hardware advancements of the initial prototype, these relate mainly to the electronic components. The proposed modifications notably bolster the repeatability of the experiments on this cost-effective device, and the careful selection of components aims to facilitate potential home assembly of the device. In addition, the software enhancements now include an expanded array of available APIs, such as Simulink, thereby simplifying the device’s integration into educational settings. Furthermore, we examine its versatility by interfacing with open-source application programming interfaces (APIs) like C/C++, Arduino IDE, Simulink, CircuitPython, and REXYGEN. By doing so, we highlight both the strengths and limitations of the MagnetoShield across various software settings and hardware configurations.

The paper also presents an unconventional model of system dynamics, particularly relevant to devices employing a permanent magnet as a levitating object. Addressing the challenges in modeling such systems compared to conventional ferromagnetic-based solutions, we thoroughly explain these intricacies, providing comprehensive insights into the device’s operation. Finally, the paper presents practical use cases that leverage more advanced control algorithms, namely model predictive control (MPC), thereby expanding its utility beyond basic experimentation towards more challenging control engineering applications.

Our intention is not to present yet another variant of this well-known experiment, but to create an open hardware and software design that allows collaboration in control and mechatronics education and even research on a budget.

## 2. Hardware Reference Design

Let us first provide an overview of functional connections, then explain the electronic and mechanical design in more comprehensive detail. The goal is to propose a reference design for the hardware, which is easily and universally replicable.

**Figure 3:**
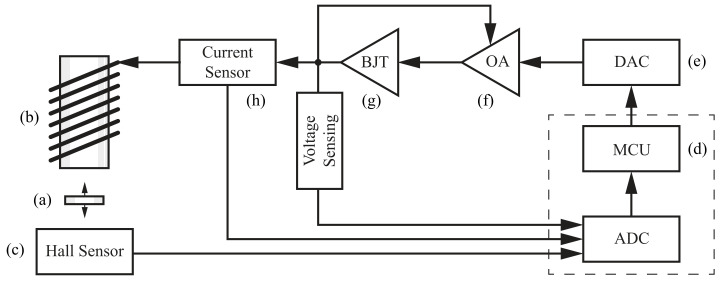
Schematic overview of the proposed magnetic levitation experimental device.

Figure 3 represents the MagnetoShield as a functional block diagram. A small permanent magnet (a) is pulled upwards by an electromagnet (b), while its position is indirectly sensed by magnetic flux density readings reported by a ratiometric linear Hall effect sensor located below (c). Provided a control algorithm running on the microcontroller (d) supplies correctly computed decision variables converted to voltages supplied to the coil based on position measurements, this will create a single-input and single-output (SISO) feedback system, ultimately tracking the vertical position of the levitating permanent magnet.

The input $u_{k}$ originating from the microcontroller is translated to a voltage signal by an external digital-to-analog (DAC) integrated circuit (e). This is consequently further amplified by an operational amplifier (f) driving a bipolar junction transistor (g). The output $y_{k}$ collected by the Hall sensor is directly supplied to the onboard analog-to-digital converter (ADC). A current sensor (h) and voltage-sensing circuitry provide additional measurements for modeling and verification purposes.

### 2.1. Electronic Circuit

**Figure 4:**
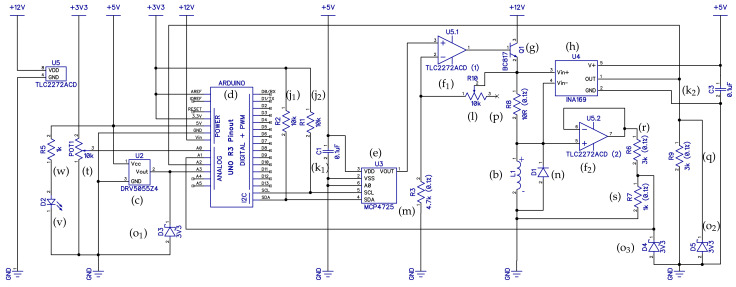
Electronic schematic diagram of the MagnetoShield.

The electronic schematic diagram of the proposed device is shown in Figure 4. In order to make our reference design accessible to students and beginners alike, we have designed the circuit and the resulting printed circuit board (PCB) in the DipTrace EDA/CAD application. The software is available in a freeware variant for simple designs such as the MagnetoShield and has a relatively gentle learning curve. To make our proposed reference hardware truly open-source, the circuit design with the final PCB is downloadable in an editable format, along with Gerber files ready for immediate manufacturing [17].

Long stackable headers (i_1_–i_3_) connect the device to the pins of an Arduino R3 layout-compatible microcontroller prototyping device from the bottom, while still leaving a female header free for connecting external devices at the top, such as oscilloscopes for monitoring the dynamic signal. The assumption of the Arduino R3 pinout serves a dual role in the hardware design. First, it establishes the physical location, pitch and count of the header pins, so that the device can be accepted by a range of compatible prototyping boards. Second, it precisely defines the electrical and logical behavior of each header pin, so that the device remains compatible with various microcontrollers even across different architectures and families.

#### 2.1.1. Input Path: Driving the Electromagnet

Let us first analyze the path of the actuation signal. The most common type of compatible MCU prototyping boards in use are of course the AVR architecture Arduinos and their third-party equivalents that are unfortunately lacking a DAC chip built into the microcontroller. Our preliminary tests have shown [18] that the relatively slow pulse-width modulated (PWM) output pre-configured in the Arduino environment is unsuitable for the fast dynamics of magnetic levitation. Thus, an external DAC ((e), U3) has been added to the inter-integrated circuit (I2C) bus at the cross-platform serial data (SDA) and serial clock (SCL) pin locations with their corresponding pull-up resistors ((j_1_), R2 and (j_2_), R1). As customary, the voltage supply of the DAC chip is filtered by a ceramic capacitor ((k_1_), C1). We have chosen a 12-bit device for increased resolution in the driving signal and powered it from the 5 V rail to scale its output to 0–5 V.

The voltage output of the DAC is then boosted by a rail-to-rail operational amplifier ((f_1_), U5.1) connected to 12 V via the VIN pin. This means that an external power supply connected to the barrel jack on the prototyping board is required to run the device. The operational amplifier is connected in a non-inverting configuration such that the feedback loop follows the voltage passing through the electromagnet ((b), L1), while its gain may be calibrated by the surface-mounted trimmer in the feedback branch ((l), R10) and a fixed resistor in the grounding branch ((m), R3). The trimmer shall be calibrated to provide 10.0 V through the electromagnet at DAC saturation, which may be achieved with a simple multimeter or an oscilloscope. This hardware design assumption is later utilized in the software interface.

A transistor ((g), Q1) powered from the high-side 12 V rail is driven through the output of the op-amp, ultimately supplying the electromagnet (b). A generic protection diode ((n), D1) is connected anti-parallel to the electromagnet in order to prevent possible damage from back electro-motive force (EMF) during transients. The electromagnet leads are connected to the underlying PCB via a corresponding pair of two pin male and female headers (i_4_) and (i_5_).

#### 2.1.2. Output Paths: Magnetic Flux Sensing, Current and Voltage Measurement

A linear ratiometric Hall effect sensor ((c), U2) connected to the A3 analog pin measures the proximity of the levitating magnet. The strongest magnetic flux ∼2000 G is measured when the magnet rests on the surface; thus, the sensor range must be selected accordingly. Unfortunately, the fairly strong maximal flux limits the selection of available components. We have chosen a bipolar device with zero flux calibrated at 2.5 V when supplied from the 5V rail; thus, a 3.3 V Zener diode ((o_1_), D3) is inserted to clip voltages that would possibly damage the built-in ADC when the magnet polarity is switched. The magnetic flux is recomputed to distance readings in software, based on an automatic calibration procedure.

The current passing through the electromagnet is an important state variable playing a role in system identification or online state estimation. An integrated high-side current monitor ((h), U4) essentially measures and amplifies the voltage drop across a precision shunt resistor ((p), R8). The current sensor gain is set by a resistor ((q), R9) placed at its analog output, while another Zener diode ((o_2_), D5) protects the ADC from possible overvoltage. A ceramic capacitor ((k_2_), C3) filters the supply of the current sensor. The output of the current sensing IC is then connected to the A2 analog pin of the microcontroller board.

The voltage measured across the electromagnet is another auxiliary output that may be useful for calibration, modeling and verification purposes. In theory, this shall be equal to the input voltage u(k) and our verifications show this holds for the purposes of this device. The voltage output is measured by a 3:1 voltage divider consisting of two resistors ((r), R6 and (s), R7), which is separated from the power circuit by another operational amplifier stage in an input buffer configuration ((f_2_), U5.2). The ADC is yet again protected from possible overvoltage by a Zener ((o_3_), D4) clamp and the signal is fed to the A1 pin of the microcontroller board.

#### 2.1.3. Additional Functions

A potentiometer/trimmer ((t), POT1) with a plastic shaft (u) is connected to the A0 analog input of the onboard ADC. The potentiometer can be used, e.g., to set the desired tracking reference manually or to tune controller parameters.

Since the shield covers the microcontroller board below, a small LED ((v), D2) powered through a resistor ((w), R5) indicates that the board is powered on.

#### 2.1.4. Logic Level and External Power Compatibility

Adapting the Arduino R3 layout does not guarantee compatibility between microcontroller architectures using different voltage levels to represent logic states. The best known MCU in this format, the ATmega 328p MCU (Arduino Uno), and others from the AVR architecture, such as the ATmega 2600 (Arduino Mega), operate using 5 V transistor-to-transistor logic (TTL) signals. On the other hand, higher performance chips from the ARM Cortex-M family (Arduino Due and Arduino Zero) adapt 3.3 V complementary metal–oxide–semiconductor (CMOS) signaling. The MagnetoShield has been designed such that it remains compatible with both logic levels and thus both dominant architectures. This is a simple matter of scaling all analog inputs sent to the built-in ADC below 3.3 V while setting an external reference for the AVR architecture MCUs by connecting the analog reference AREF pin to the 3V3 regulator.

This adjustment results not only in compatibility with the Arduino Uno, Mega and of course Zero and Due boards, but also makes it possible to use the MagnetoShield with some third-party microcontroller evaluation boards using the same, or similar MCUs as the ones above, respecting the discussed layout. A good example of the usage of the MagnetoShield with such boards can be given with the implementation of the shield with the Adafruit Metro boards, where mostly just a minor workaround regarding the timers and compiling is needed, which is discussed more in detail in Section 3.1.

On the contrary, certain third-party microcontroller evaluation boards, such as the Nucleo-series from ST Microelectronics are partly Arduino R3 pin layout compatible but lack an external barrel connector for power supply. As a 12 V rail is needed for the electromagnet, the MagnetoShield is not hardware-compatible with these prototyping tools.

#### 2.1.5. PCB Design

Since the printed circuit board doubles as a mechanical base for the electromagnet-holding bracket, we recommend a standard thickness (1.6 mm) substrate. The physical outline of the Arduino R3 layout in combination with the double-layer board using standard manufacturing tolerances allows one to benefit from the services offered by numerous low-cost PCB prototyping services.

**Figure 5:**
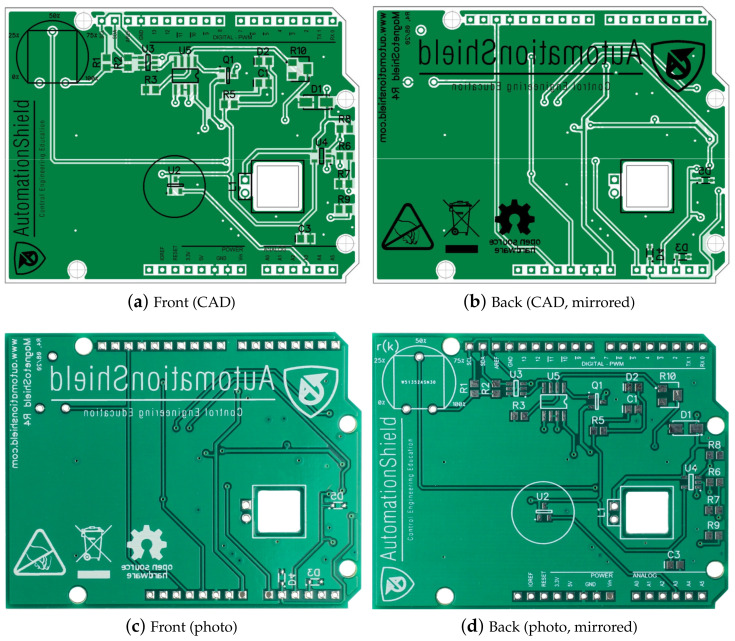
The printed circuit board of the MagnetoShield device (1:1 scale).

The EDA/CAD design of the MagnetoShield PCB is shown for the top and bottom layers in Figure 5. The editable layout file may be modified or improved or, alternatively, we also provide the Gerber files prepared for manufacturing.

**Figure 6:**
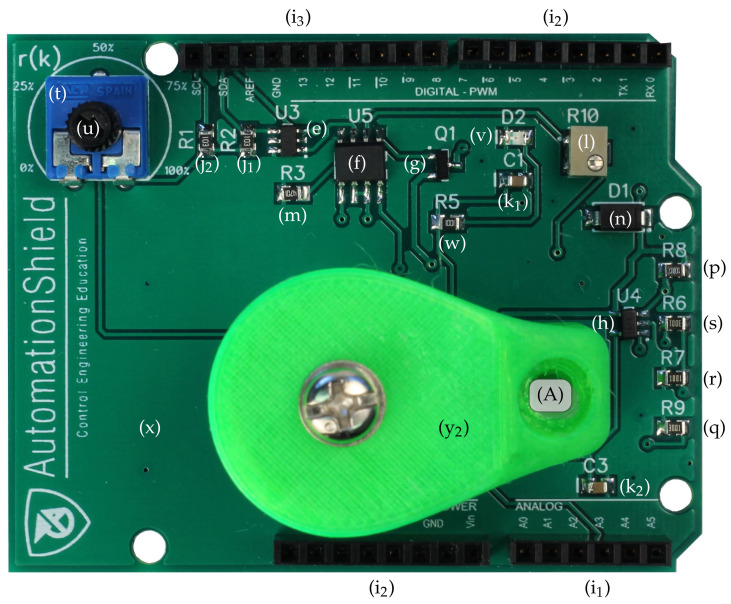
Top view of an assembled MagnetoShield (1.5× scale).

The distribution and density of components have been designed for hand-soldering. Most components are on the top layer with the exception of the Zener clamps ((o_1_)–(o_3_)). Our experience shows that the components may be hand-soldered with moderate experience, while assembling the board may be an educational experience in its own right. A top view of the finished PCB with the components soldered in their final locations is shown in Figure 6.

### 2.2. Mechanical Assembly

**Figure 7:**
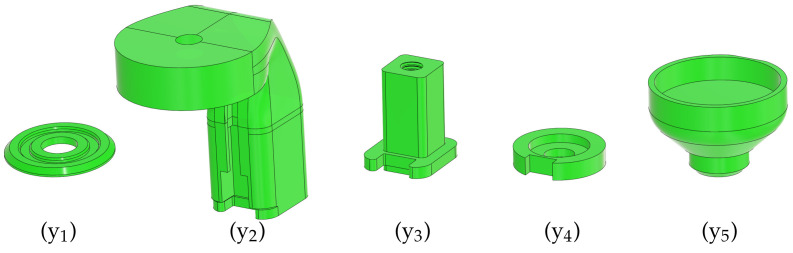
CAD renders of the 3D printed components of the mechanical assembly.

As has been mentioned before, the PCB is mechanically fixed to the underlying prototyping board via the header pins (i_1_)–(i_3_), while the rest of the superstructure holding the electromagnet is affixed to the printed circuit board (x). Figure 7 illustrates the 3D-printed plastic components.

A disc-shaped part (y_1_) surrounds the Hall sensor and it keeps the magnet flat when resting at its stable position. This part is centered manually by the circle seen on the PCB silkscreen and then glued to the surface. Commonly available cyanoacrylate glue is not recommended because of its evaporation; instead, single-component epoxy is suggested. This base holds a 10 mm high translucent plastic tube (z), which prevents the magnet from falling out of the apparatus. The main concern is that the magnet flips its polarity and remains stuck to the magnetized core of the electromagnet, which, due to the small size of the device, is difficult to remove.

The electromagnet is suspended in the air by an “L”-shaped bracket (y_2_) and is affixed to the top by a screw that comes as an accessory with the component. The bracket contains vertical grooves for the electromagnet leads and a gap to accommodate a pair of male/female header pins to connect said leads to the PCB. The bracket slides into a column with a square cross-section (y_3_), aiding the correct positioning of the magnet horizontally over the assembly. Although the bracket is held in place quite firmly by the sliding mechanism and the header pins, an M4 plastic screw (A) is inserted through the bracket to the column as well. The bottom of this column slides into a square groove that has been cut into the PCB itself; then, a washer with a corresponding square groove (y_4_) terminates the assembly from the bottom using an M5 plastic machine screw (B).

**Figure 8:**
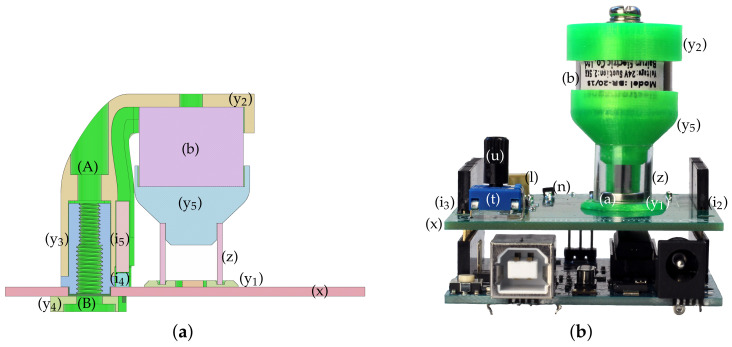
Mechanical assembly of the MagnetoShield device (1:1 scale) in a section cut (**a**) and in the front view (**b**).

The bottom of the magnet is finished by an end cap (y_5_). The cap closes the gap with the tube physically, so the magnet may only fly in an enclosed space and it cannot fall out of the apparatus during transportation. The cap creates a gap between the surface of the electromagnet and the levitating magnetic disc so that it does not stick to the magnetized inner core accidentally. A sectional cut of the assembled magnet-holding superstructure is shown in Figure 8a, and finally, Figure 8b depicts the front view of the MagnetoShield with the mechanical assembly.

We have designed this magnet-holding superstructure in AutoCAD Fusion 360 because this cloud-based software is freely available to students and hobbyists and supports efficient collaboration. Just like the circuit scheme and the PCB, the 3D mechanical design files are openly available for further improvement [17]. The components have been printed on a hobbyist-grade Prusa MK3/S 3D printer from standard ϕ = 1.75 mm PETG filament. These mechanical components may be printed in less than 2 h.

### 2.3. Parts List, Availability and Cost

Table 1 contains a complete bill of materials (BOM) for the proposed magnetic levitation device including component recommendations and prices. The parts list may be contrasted to the description above by the alphabetical markings or to the circuit diagram in Figure 4 based on the component reference designators.

Particular attention has been paid to avoiding the inclusion of exotic and hard-to-find components; thus, all parts within the list are standard and globally available. All selected electronic an mechanical components are normally available in the stock of multi-national distributors or well-known auction sites. Certain components—such as resistors, capacitors and diodes—may be swapped to compatible equivalents effortlessly. Others, especially with differing packages, e.g., the potentiometer, may be adapted by slightly modifying the PCB layout.

All prices are shown in U.S. dollars and are valid at the time of writing this article. The table and the final computed price are indicative of direct material cost and exclude ancillary expenditures such as postage, equipment maintenance, consumables, energy or even labor. The cost of the microcontroller prototyping board itself and the external 12 V wall plug adapter has been also omitted since these are reusable for other purposes as well and are widely available in the toolboxes of engineering students and the inventory of academic institutions.

The prices of components are given for two typical scenarios: (i) a student or hobbyist completing a single device for individual use or (ii) an educator or technician assembling several tens of instruments for equipping an entire teaching laboratory or classroom. In the first case, we assume that components are sourced locally and that the absolute minimization of cost is not the priority, but rather the convenience of ordering from the minimal number of part dealers. The cost of one device is in the order of the low tens of dollars, specifically under USD 35. In the latter case, we have assumed that price is more of a concern, yet procuring from several sources over a longer time period is not. After comparing parts in a component meta-search engine, we found that the unit price can be kept below USD 10. Although the ancillary costs may start to play a more significant role in the latter case, bulk deals on components or changes in technique (such as employing an SMD stencil) may offset these costs.

## 3. Application Programming Interfaces

The goal of the application programming interfaces introduced below for various languages is to aid and expedite the developing and testing of a control engineering application, instead of spending time on bridging basic sensor–actuator functionality. Nevertheless, building the control system from the grounds-up including interfacing may be a valuable learning experience in an educational setting, such as in a course aimed specifically at microcontroller technology. The role of the MagnetoShield API is then to create an abstraction layer between the hardware and the control engineering problem itself.

The hardware interface for the MagnetoShield belongs to a wider set of software called the AutomationShield API. This is essentially a collection of API, worked examples and supporting material—such as models or measurement data—for the individual devices created within this initiative.

The primary implementation target is the Arduino API, for which the source code is written in an environment-specific C/C++ dialect, referred to by some as the Arduino language. In the Arduino software ecosystem, the naming convention of functions and methods is partly unified, for example, various hardware units are initialized by the same begin() method, while outgoing signals from the prototyping board contain the word “write”, incoming “read” in their naming scheme. The nomenclature of essential functions for the MagnetoShield conforms to this, but it is also preserved between various languages and for other existing and future shields within the initiative. For example, the method actuatorWrite() remains valid for all hardware regardless of the nature of the actuator, while it works, e.g., in Arduino and Python alike. The internal logic of the API is largely identical between implementations; thus, first, we will elaborate on the Arduino IDE variant in detail and then add specifics for the Python, Simulink and REXYGEN variants.

Needles to say, the entire AutomationShield API is open-source [17], and further improvements are welcome from anyone wishing to contribute. Source control, versioning and integration is managed systematically, since the software is maintained in a public Git repository.

### 3.1. C/C++ for the Arduino IDE

**Figure 9:**
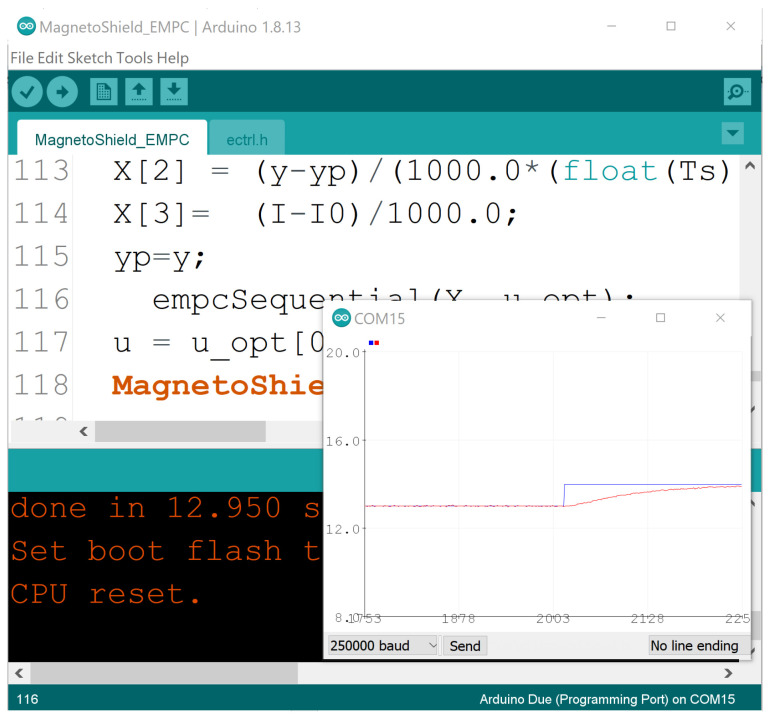
Tracking magnetic levitation reference by explicit model predictive control, showing the requested reference and measured distance in the Serial Plotter tool.

The API for the Arduino IDE (see Figure 9) is a standard library written specifically for this free development environment, and it may be installed effortlessly via the graphical user interface even by beginners. The interface to the magnetic levitation device is contained in the MagnetoShield.h header and the corresponding MagnetoShield.cpp implementation file. Including this header file in a project will create an instance of the MagnetoShield object, which contains methods to initialize the device, read various sensors and to control the electromagnet.

The library uses preprocessor directives to conditionally compile code sections to preserve compatibility between earlier MagnetoShield designs and various microcontroller architectures. The SHIELDRELEASE token is normally opaque and defaults to the latest release. The architecture and microcontroller are selected in the graphical user interface (GUI) of the Arduino IDE; then, this selection is passed to the preprocessor at compile time as a token, such as ARDUINO_ARCH_AVR. Architecture differences manifest in ADC reference voltage and resolution settings, the number of I2C buses, etc.

In case of using any compatible Arduino board, these settings will take place automatically in the background. Only for third-party compatible devices (e.g., the ones from the Adafruit Metro family) some additional steps are needed, namely: the user has to add the particular board to the GUI [19]. After that, the compiler will setup the correct configurations, thanks to the fact, the MagnetoShield library is written in a way to handle these changes too.

#### 3.1.1. Initialization and Self-Calibration

The device is initialized once at the beginning of the application by the

MagnetoShield.begin();

method, which includes the default Wire library and starts the I2C bus for various processor architectures. Since the 3.3 V rail is connected to the analog reference pin AREF for logic-level compatibility, the routine also sets the ADC reference level in software to external, thus keeping the 10-bit resolution for AVR architecture microcontrollers. The ADC resolution is increased to 12 bits for ARM Cortex-M architecture prototyping boards.

Next, self-calibration is performed by calling the

MagnetoShield.calibration();

method. The electromagnet is turned off by supplying 0 levels to the DAC and the corresponding maximum of 100 ADC readings in the Hall-effect sensor are stored in an integer variable. After waiting for transients to settle, the maximal saturation voltage is supplied to the electromagnet, thereby keeping the magnetic disc at the top of the enclosure and noting the minimum reading to filter for noise. These readings may be later accessed by the getMinCalibrated() and getMaxCalibrated() methods.

After performing magnetic flux measurements at several levitation heights of the magnet, it has been determined that the magnetic flux $B_{\left(k\right)}$ measured by the Hall sensor is related to the distance $d_{\left(k\right)}$ referenced from the electromagnet surface as

(1) $$d_{\left(k\right)}=p_{1}B_{\left(k\right)}^{p_{2}},$$

where $p_{1}$ and $p_{2}$ are calibration constants. To account for minute differences in the assembly, these parameters are computed by an automatic two-point calibration procedure. Measuring flux $B_{\max}$ when the magnet rests at the bottom of the device being the farthest from the electromagnet $d_{\max}$ and then flux $B_{\min}$ when it is the closest to the electromagnet $d_{\min}$ gives

(2) $$\begin{array}{cc} p_{1} & =\frac{\log\left(\frac{d_{\min}}{h_{\max}}\right)}{\log\left(\frac{B_{\max}}{B_{\min}}\right)}, \end{array}$$

(3) $$\begin{array}{cc} p_{2} & =\frac{d_{\max}}{{\left(B_{\min}\right)}^{p_{1}}}. \end{array}$$

The maximal voltage passing through the coil is also measured and stored in a private variable that may be accessed by the getVoltageRef() method. There are default parameters stored in the library for the distance reading function in (1) along with limit magnetic flux readings. It is known based on the calibration state stored in the calibrated variable whether the calibration function has been invoked in the application by the user, and if not, certain functions default to stored settings.

#### 3.1.2. Input

The voltage in the electromagnet is set by passing a floating point number to the method

MagnetoShield.actuatorWrite(u);

where $u_{k}$ (V) is the input ranging from 0 to 10 V. First, this voltage is scaled to corresponding 12-bit DAC levels by the voltageToDAC() private method. Because of the hardware feedback, this relationship is close to linear for our purposes. However, a slight software correction is made to compute the DAC levels $u_{\mathrm{DAC}(k)}$ by applying the

(4) $$\begin{array}{c} u_{\mathrm{DAC}(k)}=p_{3}u_{\left(k\right)}^{3}+p_{4}u_{\left(k\right)}^{2}+p_{5}u_{\left(k\right)}+p_{6} \end{array}$$

polynomial, where the coefficients $p_{3}$ , $p_{4}$ , $p_{5}$ , and $p_{6}$ are calibrated offline. The result is saturated to prevent possible overflows.

Next, the desired DAC levels are sent to the chip itself by the dacWrite() method. This accepts the 12-bit $u_{\mathrm{DAC}}\left(k\right)$ level on its input, addresses the DAC chip on the I2C bus and separates the payload into two bytes. This function is conditionally compiled based on the used MCU architecture and DAC chip to preserve compatibility with earlier designs.

Inputs can be expressed in percentual units relative to the maximum coil voltage as well by invoking the actuatorWritePercents() method. This re-scales them to input voltage based on the maximum calibrated values measured when the device was initialized.

#### 3.1.3. Output

Although feedback control design is possible with physical units that are merely related to the position of the magnet, first-principle and then later grey-box system identification and the resulting state feedback are more engaging in direct distance units.

Therefore, at first, the adcToGauss() method reads the ADC levels from pin A3 based on conditional compiling considering the correct ADC resolution and reference voltage, then returns the flux contingent on the gain and bias supplied by the component manufacturer. For the Hall sensor considered in this reference design, the zero is at 2.5 V for a 5 V supply and its sensitivity is 1.25 mV/G. The returned flux is re-mapped to distance units by the gaussToDistance() method using (2) and the two calibration parameters obtained at startup.

From the viewpoint of the user, the API simplifies this to

y=MagnetoShield.sensorRead();

which returns the distance $y_{\left(k\right)}=d_{\left(k\right)}$ (mm) of the permanent magnet from the electromagnet as a floating point number.

Percentual position readings are accessible through the sensorReadPercents() method, based on the minimal and maximal flux readings gathered at calibration, while the magnetic flux $B_{\left(k\right)}$ (G) is also available by calling sensorReadGauss().

#### 3.1.4. Auxiliary Functions

Most mathematical models of magnetic levitation applicable to this scenario—especially those considering internal states—utilize the current passing through the coil of the electromagnet as a state variable. The current $i_{\left(k\right)}$ (mA) may be accessed by calling the

i=MagnetoShield.auxReadCurrent();

method, returning a floating-point number. This method recomputes the ADC levels on pin A2 to voltage, then shifts it by a linear bias and scales the result by a gain provided by the manufacturer of the current sensing IC.

Similarly, the voltage potential measured at the electromagnet $v_{\left(k\right)}$ (V) on A1 is available using the

v=MagnetoShield.auxReadVoltage();

method, returning a floating point variable. The ADC levels are converted to voltages and then corrected by the 3:1 gain of the impedance-buffered voltage divider. As has been pointed out earlier, while the circuit itself renders this largely equal to the desired input $u_{k}$ supplied to the system, the measurements may be utilized to calibrate a correction curve.

Finally, the position of the onboard potentiometer at the pin A0 may be acquired by calling

r=MagnetoShield.referenceRead();

returning the reference $r_{\left(k\right)}$ (%) as a floating point variable. This external reference supports interaction with the device by allowing for manual tracking, even though it may be reprogrammed to an alternative role such as controller tuning.

#### 3.1.5. Common API Modules

The AutomationShield API contains several common modules supporting the formulation of feedback control applications. For example, establishing a framework for hard real-time sampling using hardware timers and interrupts could be a daunting task for beginners. The Sampling subsystem included in the API implements a simplified framework across several compatible MCU architectures. Others provide the means for PID control, Kalman filtering and others.

### 3.2. Python API

Python is constantly gaining traction as the programming language of choice for many engineering areas; however, it is not traditionally thought of as suitable for embedded applications.

The reason is that it is an interpreted language, which makes efficient memory management and hard real-time operation practically impossible. Also, lower-tier microcontrollers cannot run Python code, since the interpreter has to reside on the chip itself, and what is more, it has to be a hardware-specific interpreter implementation. Nevertheless, Python, when compared to C/C++, increases readability, expressiveness and productivity [20]. Despite these limitations, it is possible to run Python code on embedded microcontrollers and other limited systems even for feedback control. MicroPython offers hardware-specific implementation of a subset of the Python language and standard libraries to a handful of ported microcontrollers [21], while CircuitPython is its education- and experimentation-friendly open-source derivative [22]. The transition from full-fledged Python to Micro- or CircuitPython is effortless; however, the interpreted nature of the language [23] should be taken into account. Since the main motivation in creating and maintaining the proposed MagnetoShield device and supporting software is to help students and educators, we have chosen to write an API in CircuitPython. At the time of the writing of this article, only a handful of references mention CircuitPython in the literature [24,25], while none demonstrate its utilization for feedback control experiments.

The MagnetoShield API is contained in the MagnetoShield module that shall be imported in the user application. The module itself imports further libraries to access hardware functionality, timing and basic mathematics. After including the API module, identical nomenclature is valid for the Python application as for the Arduino IDE. That is, the input is sent to the electromagnet by MagnetoShield.actuatorWrite() and its position can be read by MagnetoShield.sensorRead(), etc. The reader shall refer to the description given for the Arduino API for a complete list of available functions. The internal structure and workings of the Python API remains as close to the Arduino counterpart as possible, except for the CircuitPython and hardware-specific calls.

**Figure 10:**
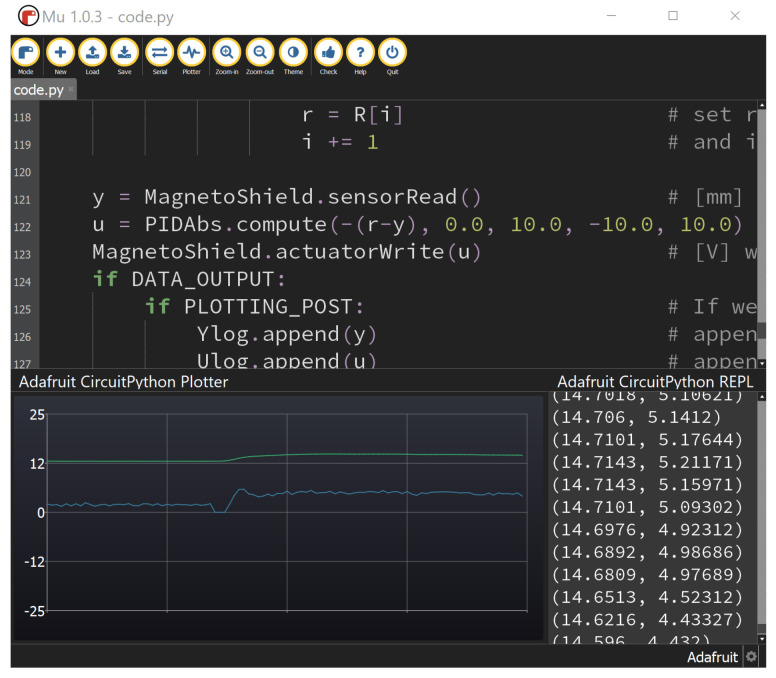
CircuitPython source code for the PID control of magnetic levitation showing the graphical output of input voltage and distance in the Mu editor.

The CircuitPython source code for the given control application does not even require a dedicated IDE and may be edited by any standard text editor and uploaded to the prototyping board which is handled as a drive by the computer. Saving the code online, accessing the interpreter directly by the read evaluate print loop (REPL) and displaying live measurements in a graphical output in a free open-source editor such as Mu is also an option; see Figure 10.

Similar to the Arduino API, the Python variant offers certain supporting functions for sampling, PID control, etc.

#### Limitations

As expected, hard real-time sampling is not achievable. Since Python is an interpreted language, it has much less predictable performance than compiled languages, such as C/C++, which is routinely used for embedded systems. The sampling of feedback control may be unfortunately affected by the memory management mechanism known as “garbage collection” and background tasks such as USB activity and file operations.

Certain MicroPython ports implement access to hardware timers, but CircuitPython on Arduino R3-compatible prototyping boards currently does not offer this option. The Sampling subsystem considered for the MagnetoShield Python API relies only on a built-in monotonic timer counter, storing its result in a floating-point number. As a consequence, timer resolution decreases as time passes. Moreover, sample timing is prone to jittering because of the interpreted nature of the language and the issues mentioned above.

Assuming simple control algorithms, such as PID or LQ with direct state measurement, the powerful Microchip ATSAMD51 Cortex M4 microcontroller clocked at 120 MHz just barely manages to stabilize the system, while the mid-range Microchip ATSAMD21G18 Cortex M0 MCU running at 48 MHz shows visible degradation in tracking quality.

There are some constraints for data logging; thus, it is recommended to send measurements to an outside terminal program instead of the Mu editor since its graphical plotter cannot keep up with the data rate in real time. While this may be circumvented by storing measurements in the internal memory on more powerful models, the M0 family MCU will not be able to output data to REPL and control magnetic levitation concurrently.

### 3.3. Simulink API

Thanks to its graphical programming interface and code-generation capability, Simulink remains a popular choice for educators and professionals as well. Its prime intention has long moved from modeling and simulation to the realm of direct code deployment and physical computing.

**Figure 11:**
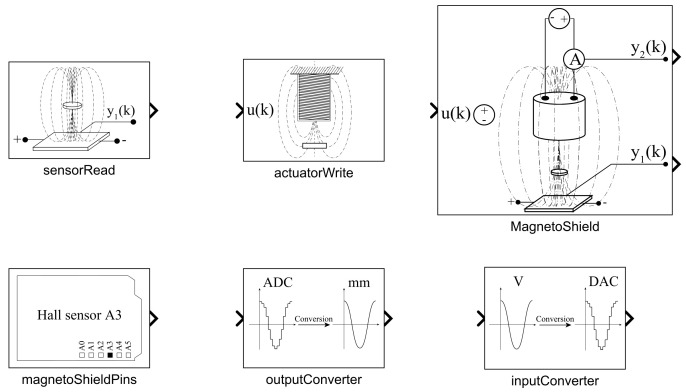
The collection of Simulink blocks constituting the MagnetoShield API.

To reflect this, a collection of blocks serving as the API for the MagnetoShield was created, which are illustrated in Figure 11. Combining the hardware and the simplified API introduced here with the enormous selection of advanced signal processing and control algorithmic blocks creates a powerful learning environment.

The Simulink API for the MagnetoShield requires the Simulink Support Package for Arduino Hardware to access the physical computing platform itself. The block scheme is transcribed to a C/C++ source code and then compiled and directly deployed to the target microcontroller. The application may run stand-alone or tethered to the development computer, where features such as transferring data to virtual oscilloscopes and handling switches and sliders are taken care of. Currently, the Simulink Support Package for Arduino Hardware is compatible only with the Arduino Uno, Mega2560 and Due models. The MagnetoShield Simulink API handles differences between hardware and architectures by allowing the user to select the target board inside blocks. The internal logic of the algorithmic blocks is largely identical to the previously introduced API variants, and therefore will not be repeated here.

Since the default choice of Simulink signal type is a double floating-point number, we have chosen to preserve this for consistency and ease of use. The blocks are configurable via a graphical dialog box with settings such as the sampling rate, target MCU, or signal units.

#### 3.3.1. Outputs

Similarly to the Arduino API, acquiring a distance reading of the flying magnet is integrated into a single program unit, called the sensorRead block. Here, the user may select the target device then a desired reading from the onboard sensors: magnet distance, coil current or voltage and reference from the potentiometer. This block includes functionality equivalent to the sensorRead(), auxReadCurrent() and auxReadVoltage() methods in the Arduino and CircuitPython variants of the API.

Those wishing to have more control over the direct output and auxiliary sensor readings may alternatively access the ADC levels at different pins directly by the MagnetoShieldPins block. This reads the levels as 16-bit unsigned integers and then converts them to a double-precision floating-point number. The graphical representation of the block changes according to the selected input type.

The ADC levels may be then converted to physically meaningful units by employing the OutputConverter block. Here, the target board and the desired measurement and its units, such as position, voltage, current and percentage, may be selected. Fine-tuning the conversion is also possible by, e.g., re-defining the parameters of (1) or choosing the voltage and current measurement gain.

#### 3.3.2. Input

Choosing the actuatorWrite block from the Simulink API is sufficient to send inputs to the electromagnet. Again, it is possible configure what type of signal is expected by the block: voltage, percentual values or direct DAC levels. This signal is internally converted to a 16-bit unsigned integer, then passed to the DAC chip via the I2C bus. In essence then, this block replaces the actuatorWrite(), actuatorWritePercents() and dacWrite() methods introduced earlier for the C/C++ and Python versions of the API.

Should tighter control over the inputs be required, the inputConverter block provides this option. The GUI allows switching between voltage, percentual mapping or direct DAC levels.

#### 3.3.3. Full MagnetoShield Hardware Access

The simplest means to create a feedback control application for the proposed device in Simulink is the MagnetoShield algorithmic block, which unites input and output functionality in a single entity. One may select between output in distance units or percents and the block reads both measurable internal states for feedback control. Internally, the block performs a calibration routine after startup, which may be disabled by a checkbox.

#### 3.3.4. Limitations

Simulink does not contain a block to switch analog reference levels to external; therefore, this must be manually configured in the simulation properties for each example. The target platform, sampling rate, solver, etc., must also be carefully configured for each example; nevertheless, fully functional and pre-configured examples are included in the library.

### 3.4. REXYGEN API

Similarly to Simulink, REXYGEN makes it possible to program embedded control hardware graphically, i.e., without hand coding. Because in comparison to the other API REXYGEN is less known, let us describe its basic architecture. The cornerstone of the implementation is the runtime core of the REXYGEN system called RexCore. This core runs on top of the operating system, coordinates algorithm execution and task scheduling, and provides access to the physical input and output signals of the deployment platform of choice through the modular system of REXYGEN I/O drivers [26]. In addition to the similarity of the environment and workflow to Simulink, a project created in REXYGEN may be tested directly in Simulink before deployment on the embedded target by the RexLib Simulink add-on.

#### 3.4.1. MagnetoShield in RexDuino

We have selected the well-known Raspberry Pi (RPi) single-board computer from the wide array of natively supported target devices in order to demonstrate the capabilities of the MagnetoShield in combination with the REXYGEN environment. Since the MagnetoShield is not directly hardware-compatible to the Raspberry Pi, we have connected the Raspberry Pi to an Arduino UNO in a setup referred to as the RexDuino [27]. In this configuration, all the control algorithms run in real-time on the Raspberry Pi (master part), and the Arduino only hosts the communication services (slave part).

**Figure 12:**
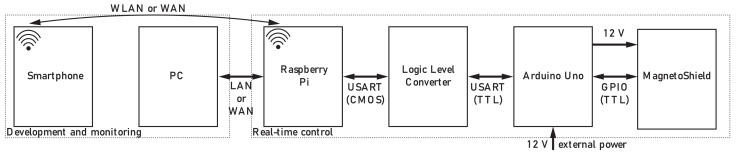
Block scheme of testing setup and its wiring diagram.

This setup is conceptually illustrated in Figure 12. The REXYGEN project is developed on a host PC and then deployed to the Raspberry Pi running the real-time control application. In turn, the RPi accesses the MagnetoShield hardware via the physical interface provided on the Arduino UNO. Note that the two boards communicate via UART; thus, logic levels must be matched from and to 5 V TTL and 3.3 CMOS for signal compatibility. Besides development, the host PC can be used for diagnostics and visualization, while a smartphone or tablet may also be utilized for process visualization.

#### 3.4.2. Feedback Control

The feedback control project has been created in REXYGEN STUDIO and then compiled and uploaded onto the RPi. In the case of the MagnetoShield, the API consists of a library containing different blocks performing key functionality needed for feedback control.

**Figure 13:**
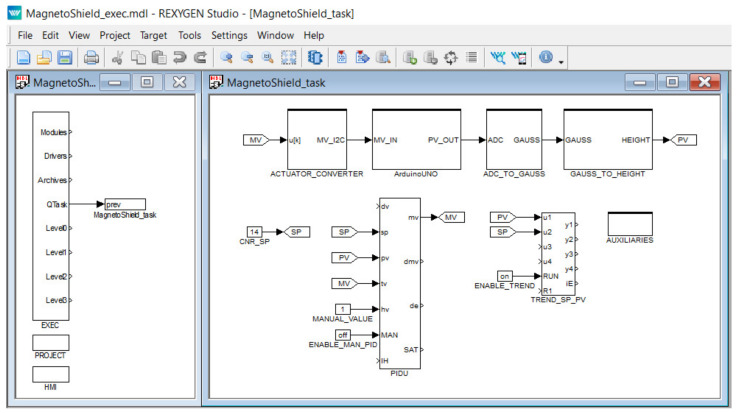
A REXYGEN project implementing MagnetoShield PID control.

Generally, a project in REXYGEN consists of at least two compulsory parts: real-time executive configuration and one or more connected tasks. Figure 13 shows an example REXYGEN project controlling the levitation height of the MagnetoShield.

The real-time executive configuration is displayed on the left (MagnetoShield_exec.mdl), showing the EXEC block used for storing the main settings of the real-time executive together with the PROJECT block defining dependencies and the HMI block with visualization settings. The right side of Figure 13 demonstrates how individual blocks are chained in case of a PID control loop. The blocks can be used separately for open-loop testing and diagnostic purposes. The closed-loop example starts with the setpoint (SP) that enters PIDU block containing a collection of PID algorithms, generating the manipulated parameter (MP) that is processed by the rest of the blocks. The manipulated parameter is converted to an I2C command for communicating with the DAC (ACTUATOR_CONVERTER), and then it enters the ArduinoUNO block containing the settings of the Arduino UNO board. This provides an integer representation of the process value (PV) that has to be converted to units of magnetic induction (ADC_TO_GAUSS), then comes the conversion to height (GAUSS_TO_HEIGHT). The trends of variables are processed by the TRND block (TREND_SP_PV), essentially allowing real time plotting and export.

#### 3.4.3. A Web-Interface for Remote Experiments

The REXYGEN environment offers highly customizable visualization tools, making it ideal for implementing remote experiments.

The dynamic part of the application contains special blocks such as sliders and buttons, while the graphical interface is created in the REXYGEN HMI Designer. Apart from the special blocks, the visualization is also composed of standard blocks (static objects) that have their properties linked to the variables from the real-time executive.

**Figure 14:**
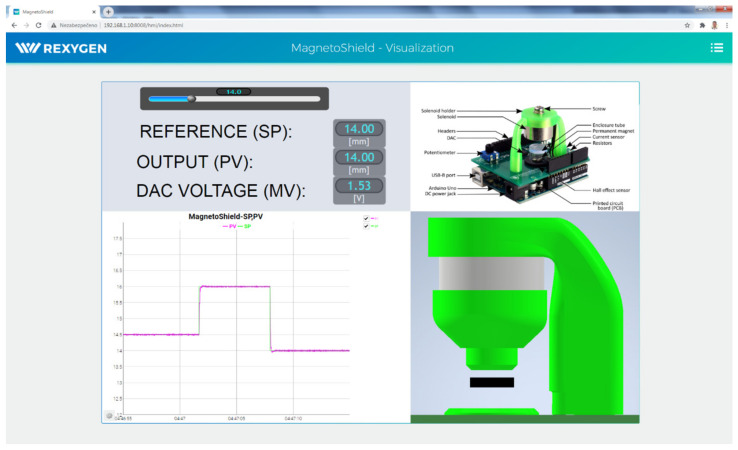
A custom REXYGEN visualization of the MagnetoShield control.

Figure 14 shows an example visualization created for the MagnetoShield. The setpoint is defined via the horizontal slider. All key variables of the control circuit (SP, PV, MV) may be displayed in the form of a numeric value or a real-time plot. The position of the levitating magnet—represented by the black rectangle—is changing dynamically over time according to its real measured value. Figure 14 is a screenshot of the visualization GUI accessed via a web browser, representing the magnet position tracking reacting to the change in setpoint at three different values.

The visualization part of the project runs on the embedded target itself using a simple lightweight built-in web server. The entire GUI may be accessed via a standard web browser without installing any external software components.

### 3.5. Too Slow to Implement: MATLAB, Octave, Scilab and LabView

The MATLAB interpreted language is widely accepted in scientific and engineering circles and, because of the high-level code and advanced specialized toolboxes, it is an ideal choice for control engineering education and research. Several open-source alternatives, such as GNU Octave or Scilab, bid to offer similar functionality free of cost. In the category of graphical programming environments, the LabView suite is also a universally recognized tool for educational institutions and research laboratories, while Xcos offers an open-source alternative to Simulink. All of the programming environments mentioned above support communication with Arduino hardware.

However, neither of these environments compiles stand-alone code directly to the microcontroller. Instead, they use a server application that communicates with the host computer via the serial interface. Despite of the fact that this setup is inadequate for hard real-time sampling, feedback control would still be possible at least for didactic applications. Unfortunately, the relatively short sampling period of $T_{s}=$ 5 ms necessitated by the dynamics of magnetic levitation and the communication bottleneck makes an implementation of an API for the MagnetoShield impossible.

**Figure 15:**
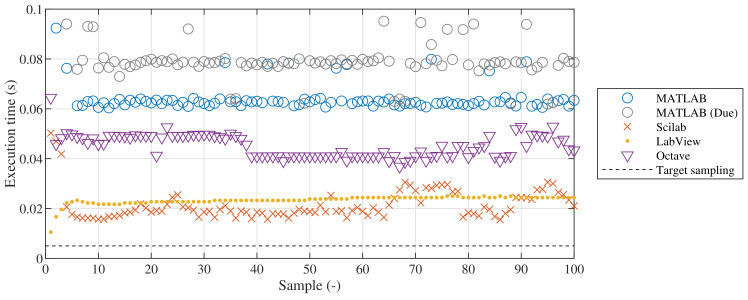
Minimal achievable sampling speeds for reading the ADC and writing to the I2C bus.

Figure 15 provides an overview of minimal achievable sampling speeds for the MagnetoShield when the application only consists of reading the ADC and writing to the I2C bus, as these would be necessary to implement for any SISO control algorithm. Despite showing almost five-fold differences for the same task, neither environment meets the target sampling rate. The average and maximum execution times are listed for various programming environments supporting Arduino boards are listed in Table 2. The testing scripts.

## 4. Demonstration Examples

The hardware of the MagnetoShield itself does not fundamentally limit the gamut of examples that may be implemented for teaching or research purposes. The fast, nonlinear and unstable nature of magnetic levitation enables users to explore challenging practical problems in control and mechatronics. The limiting factor is rather the MCU board from the viewpoint of (i) computational power, (ii) volatile memory and (iii) non-volatile memory. In addition to that, the (iv) implementation language can introduce some environment-specific constraints; especially in the case of interpreted programming frameworks such as CircuitPython.

The aim of the following discussion is three-fold: to demonstrate the functionality and closed-loop properties of the proposed hardware, to illustrate a selection of some typical algorithms covered by control engineering courses on the MagnetoShield, and finally, to show the practical limitations of microcontroller-based feedback control of this phenomena.

### 4.1. Modeling and Identification

The mathematical–physical description, system identification, model verification and simulation of the MagnetoShield presents a distinctive category of problems in mechatronics and is also necessary for the design of model-based control. Unlike most magnetic levitation devices in the literature, the MagnetoShield makes use of a permanent magnet, instead of a ferromagnetic object. Because of this, first, we shall derive a mathematical description of magnetic levitation specific for this case.

#### 4.1.1. First-Principle Modeling

**Figure 16:**
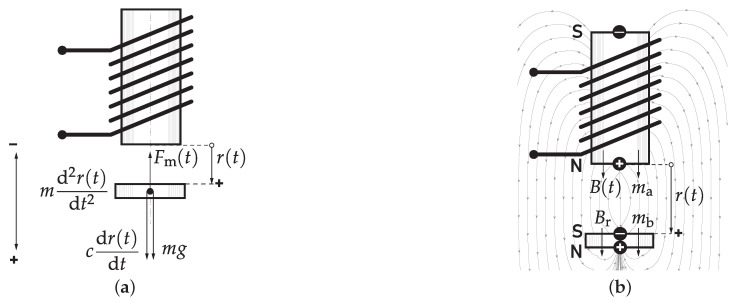
(**a**) Dynamic forces acting on the permanent magnet. (**b**) Representation of the magnetic fields for the MagnetoShield.

When the permanent magnet levitates in its unstable equilibrium, the sum of all forces acting on it is zero; thus, $F=F_{g}+F_{m}+F_{v}=0$ , where $F_{m}$ are magnetic forces, $F_{g}$ is gravitational force and $F_{v}$ is a damping force. Assuming the damping force affecting the levitating body can be modeled by viscous damping phenomena, the force is proportional to speed by the constant *c*. We may express the equation of motion along the levitation axis by

(5) $$m\frac{d^{2}r(t)}{dt^{2}}=-mg+F_{m}(t)-c\frac{dr(t)}{dt},$$

where r(t) is the time-dependent distance of the levitated permanent magnet from the surface of the electromagnet, *m* is its weight and *g* is the gravitational constant. Furthermore, we will assume that movement down and towards to the ground is the positive direction. Figure 16a illustrates the orientation of the coordinate system and the forces acting on the moving object. To date, the equation above is valid for the customary steel ball and the permanent magnet as well, but in our case, differs on the nature of magnetic force.

To keep the model simple enough for model-based control, we may approximate this magnetic force by assuming that the field of both the electromagnet and the permanent magnet can be represented by an elementary magnetic dipole. In reality, the complex interaction of the magnetic fields of the solenoid and permanent magnet depends on three dimensional geometry and would yield an unnecessarily large model for the purposes of feedback control. Note that in order to the article readable, we will not stick to the mathematical formalism of (electro)magnetic phenomena.

The time-dependent magnetic moment m(t) of a dipole according to the Ampèrian model is given by $m\left(t\right)=i\left(t\right)S\hat{r}$ , where i(t) is the current passing through a conductor and *S* is its area and $\hat{r}$ is a unit vector perpendicular to the current loop [28,29,30]. Let us denote the moment of the dipole representing the electromagnet by the lower index _a_ and then compensate for the number of windings *N* and reduce the equation to one direction along the levitation axis as [30,31]

(6) $$\begin{array}{c} m_{a}\left(t\right)=Ni\left(t\right)S, \end{array}$$

with the orientation as given in Figure 16b.

Now we shall define the constant magnetic moment of the permanent magnet, modelled as a dipole. Magnetization $\vec{M}$ or, in the other words, the magnetic moment for a unit volume V is defined by $\vec{M}=d\vec{m}/dV$ [28,32,33]. Based on Maxwell’s equations, we may simply relate flux density to magnetization at zero external field strength by the permeability of free space [28,33,34]. Thus, after denoting the permanent magnet by _b_, expressing the equation in a single direction along the levitation axis, and assuming the Sommerfeld notation for the relationship of flux density and field strength, we obtain [33,34]

(7) $$\begin{array}{c} m_{b}=MV=\frac{1}{\mu_{0}}B_{r}V, \end{array}$$

where the constant $B_{r}$ is residual flux density or magnetic remanence defining the magnetic field a material preserves once the external source is removed, while $\mu_{0}$ is the permeability of free space. Remanence essentially defines the strongest theoretical magnetic field a permanent magnet can produce at zero gap.

The force acting between the electromagnet and permanent magnet is then approximated by the force interaction between the two elementary dipoles, given by its scalar form reduced only to the levitation axis [35,36,37]

(8) $$\begin{array}{c} F_{m}\left(t\right)=-\frac{3\mu_{0}m_{a}\left(t\right)m_{b}}{2\pi r{\left(t\right)}^{4}}. \end{array}$$

Let us now substitute for the magnetic moment of electromagnet from Equation (6) and the permanent magnet from Equation (7) and then summarize the product of all remaining constants in *K* to obtain

(9) $$\begin{array}{c} \frac{d^{2}r(t)}{dt^{2}}=g-\frac{Ki(t)}{mr{\left(t\right)}^{4}}-\frac{c}{m}\frac{dr(t)}{dt}, \end{array}$$

where

(10) $$\begin{array}{c} K=\frac{3}{2}\frac{SNVB_{r}}{\pi }. \end{array}$$

**Figure 17:**
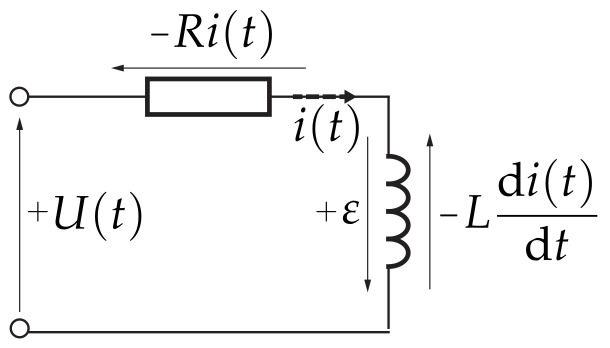
Illustration of the voltage balance.

Next, let us assume that from an electrical viewpoint, our device can be approximated by a series LR circuit. The voltage U(t) across the electromagnet then consists of ohmic resistance and the change in current in the inductor,

(11) $$\begin{array}{c} U(t)-Ri(t)-L\frac{di(t)}{dt}+\varepsilon =0, \end{array}$$

where *R* is the resistance of the electromagnet and *L* is its inductance. The final term on the right ε (V) is the effect of the moving permanent magnet on the electromagnet, contributing to the overall voltage balance. The voltage balance is illustrated in Figure 17. The polarity of ε depends on the chosen orientation of the coordinate system, and for our case, will induce voltage as moves closer to the electromagnet. Models of similar magnetic levitation devices often completely ignore this effect (e.g., [38,39]), or approximate it by adjusting the inductance of the coil in distance-dependent terms (e.g., [6,40,41,42]), while some simply take a proportional relationship between voltage and current (e.g., [43]) However, even a ferromagnetic steel ball moving in a changing magnetic field affects the voltage in the coil, the ramifications of using a permanent magnet in our design makes this effect more pronounced.

According to Faraday’s law, this motional electromotive force induced by the changing magnetic flux is [28,31,33,44]

(12) $$\begin{array}{c} \varepsilon =-\frac{d\Phi (t)}{dt} \end{array}$$

where Φ(t)=B(t)S defines that magnetic flux is magnetic induction (flux density) B(t) over an area *S* of loop wire [28,33,37]. Substituting this definition to (12) and compensating for the number of windings in our electromagnet then yields

(13) $$\begin{array}{c} \varepsilon =-NS\frac{dB(t)}{dt}. \end{array}$$

Although the flux of the permanent magnet could be calculated specifically for the disk-shaped magnet considered in the MagnetoShield, in order to keep the representation simple, let us approximate the magnetic flux density of the permanent magnet by an ideal dipole. The flux density in a distance r(t) along the axis of displacement is reduced to [45,46,47]

(14) $$\begin{array}{c} B\left(t\right)=\frac{\mu_{0}2m_{B}}{4\pi r{\left(t\right)}^{3}}=\frac{\mu_{0}m_{B}}{2\pi r{\left(t\right)}^{3}}, \end{array}$$

which, after differentiating with respect to time, yields

(15) $$\begin{array}{c} \frac{dB(t)}{dt}=\frac{d}{dt}\left(\frac{\mu_{0}m_{B}}{2\pi r{\left(t\right)}^{3}}\right)=-\frac{3\mu_{0}m_{B}}{2\pi r{\left(t\right)}^{4}}\frac{dr(t)}{dt}. \end{array}$$

Substituting for the magnetic moment of the permanent magnet from (7) back to (13) will give

(16) $$\begin{array}{c} \varepsilon =\frac{3SNB_{r}V}{2\pi r{\left(t\right)}^{4}}=K\frac{1}{r{\left(t\right)}^{4}}\frac{dr(t)}{dt}. \end{array}$$

where the constant *K* is actually the same as in (10). The final form of the electrical equation will be

(17) $$\begin{array}{c} \frac{di(t)}{dt}=-\frac{R}{L}i\left(t\right)+\frac{1}{L}U\left(t\right)+K\frac{1}{Lr{\left(t\right)}^{4}}\frac{dr(t)}{dt}. \end{array}$$

The final model of the MagnetoShield is expressed by the combination of (9) and (17). The model may be rewritten in the state-space for natively nonlinear control and estimation algorithms by choosing state variables as x(t) = ${\left[\begin{array}{c} x_{1}\left(t\right),x_{2}\left(t\right),x_{3}\left(t\right) \end{array}\right]}^{⊺}$ = ${\left[\begin{array}{c} r\left(t\right),\dot{r}\left(t\right),i\left(t\right) \end{array}\right]}^{⊺}$ and assuming the input u(t)=U(t) , yielding

(18) $$\begin{array}{cc} {\dot{x}}_{1}\left(t\right) & =x_{2}\left(t\right),\\ {\dot{x}}_{2}\left(t\right) & =g-K\frac{x_{3}\left(t\right)}{mx_{1}{\left(t\right)}^{4}}-cx_{2}\left(t\right),\\ {\dot{x}}_{3}\left(t\right) & =-\frac{R}{L}x_{3}\left(t\right)+\frac{1}{L}u\left(t\right)+K\frac{1}{Lx_{1}{\left(t\right)}^{4}}x_{2}\left(t\right). \end{array}$$

To linearize this model, we will assume that the permanent magnet will levitate around the equilibrium point $x_{01}$ , $x_{02}$ , and $x_{03}$ with $u_{0}$ . Since the magnet shall not move in its equilibrium $x_{02}=0$ (m/s) and the rest of linearization states may be chosen or experimentally measured, we may express the deviation from this linearization point as $\Delta \vec{x}\left(t\right)$ = ${\left[\begin{array}{c} \Delta x_{1}\left(t\right),\Delta x_{2}\left(t\right),\Delta x_{3}\left(t\right) \end{array}\right]}^{⊺}$ = ${\left[\begin{array}{c} \Delta r\left(t\right),\Delta \dot{r}\left(t\right),\Delta i\left(t\right) \end{array}\right]}^{⊺}$ and expand (18) into a Taylor series to obtain

(19) $$\left[\begin{array}{c} \Delta {\dot{x}}_{1}\left(t\right)\\ \Delta {\dot{x}}_{2}\left(t\right)\\ \Delta {\dot{x}}_{3}\left(t\right) \end{array}\right]=\left[\begin{array}{ccc} 0 & 1 & 0\\ \frac{4Kx_{03}}{mx_{01}^{5}} & -c & -\frac{K}{mx_{01}^{4}}\\ 0 & \frac{K}{Lx_{01}^{4}} & -\frac{R}{L} \end{array}\right]\left[\begin{array}{c} \Delta x_{1}\left(t\right)\\ \Delta x_{2}\left(t\right)\\ \Delta x_{3}\left(t\right) \end{array}\right]+\left[\begin{array}{c} 0\\ 0\\ \frac{1}{L} \end{array}\right]\Delta u\left(t\right).$$

#### 4.1.2. Identification Experiment and Parameterization

The mathematical–physical modeling outlined above may be used as a theoretical starting point for teaching practical concepts of experimental design and grey-box identification to engineering students. The AutomationShield library contains worked examples for experimental data acquisition by the MagnetoShield (MagnetoShield_Identification.ino). As the system is open-loop unstable, the experiment is carried out under PID control. Although this may raise concerns about the causality of the input–output data, these issues do not pose a practical obstacle for parameter identification, yet may be still explored in a more advanced course. The identification experiment may be carried out and logged to a data file by several open-source tools. A reference measurement is also included in the library (MagnetoShield_ID_Data.mat).

**Figure 18:**
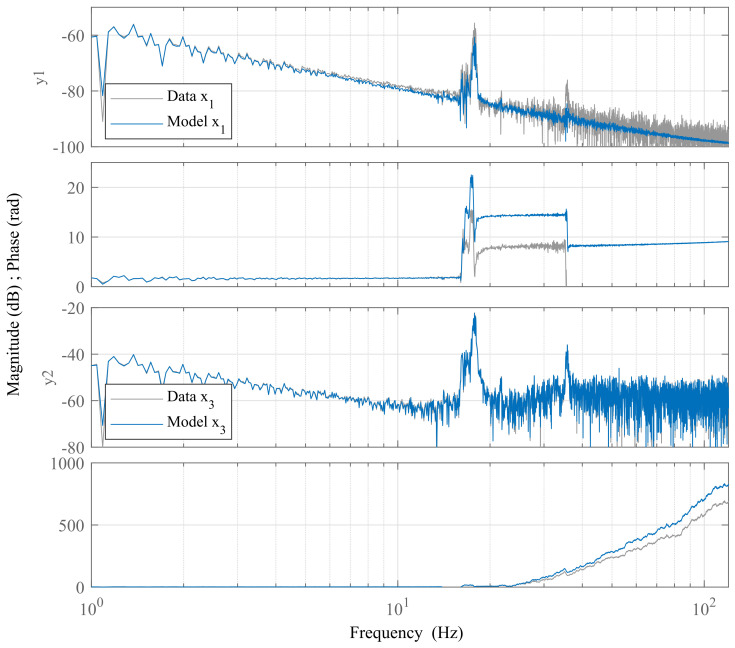
Comparison of the response of linearized model with the experimental data.

The grey-box identification of the unknown parameters is implemented in MATLAB utilizing the System Identification Toolbox (MagnetoShield_ID_GreyBox_SS.m). The model has been initialized with most parameters directly measured (see Table 3). The identification procedure then turns time-domain data to frequency-domain and finds the unknown parameters for the linearized model. Constraining identical parameters is currently not supported in the System Identification Toolbox, therefore, unlike in the physical interpretation, the constants *K* diverge in the identified model. Since the identification remains nonlinear even when using a linear model, parameters may even converge to values that are physically not meaningful. Since the damping constant *c* converged to small but negative values, we have decided to discard it from the identification and assume c=0 . The comparison of initial guesses and final parameters in a physical sense are given in Table 3. The resulting linearized model matches measurement data with 95.4 % for the position coordinate and with 97.1 % for the current. The comparison of experimental data to model response for the linearized model is shown in Figure 18 in the frequency domain.

Identifying an inherently unstable nonlinear model from experimental time-domain data is not a trivial process; thus, instead, we have chosen to simply substitute the identified physical parameters from the linear to the nonlinear model. A way to illustrate the validity of the proposed model and identification procedure is to perform a closed-loop simulation. The standard linearized model from the literature (e.g., [6,40,41]) yields an unstable response in the time-domain under PID control with matched tuning parameters.

**Figure 19:**
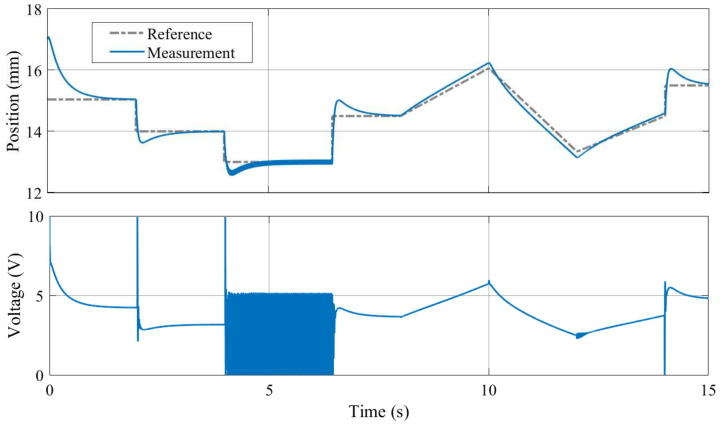
Simulation with the proposed nonlinear model using the same PID parameter tuning as used in experiments.

**Figure 20:**
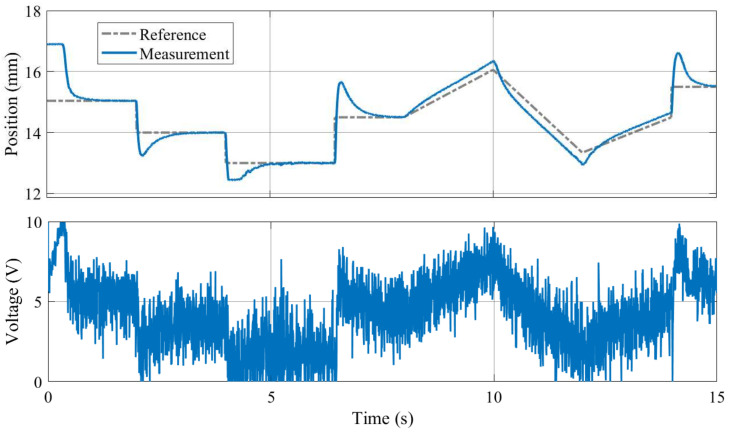
Experiment with PID tracking of levitation height.

As illustrated in Figure 19, both the proposed nonlinear and the linearized model taking the magnetic nature of the levitated disc into account is stable and represents the observed dynamics of the MagnetoShield well. For example, the reader should note the marginally unstable oscillating behavior in the output and even more dominantly in the input. This results from the nonlinearities increasing in effect as the permanent magnet is farther away from the solenoid and corresponds well to the experiments; see Figure 20.

### 4.2. Feedback Control

In addition to the API, the AutomationShield library contains numerous worked examples with detailed in-code commentary for various feedback control methods for the MagnetoShield. These currently include proportional–integral–derivative (PID), pole placement (PP), linear quadratic (LQ) and model predictive control (MPC). If possible, corresponding examples are given as offline simulations in MATLAB, and as physical experiments in C/C++, Simulink and CircuitPython. This way, students may compare the practical realization and deployment of standard control methods in different programming environments. An overview of the demonstration examples in relation to deployment methods is given in Table 4.

These examples have not only been tested across various programming environments but for different commonly available microcontroller prototyping boards as well; see Table 5. While traditional feedback methods such as PID and approaches known as “modern control” (e.g., LQ) are fully functional even on the omnipresent Arduino Uno, hardware-intensive MPC requires boards with higher processor and memory specifications. Also, the CircuitPython interpreter requires microcontroller-specific porting; thus, is only compatible to a select group of hardware. There is an enormous selection of microcontroller prototyping boards that are certainly compatible to the MagnetoShield; however, providing a comprehensive compatibility list is outside the scope of our discussion.

Table 6 lists the recommended minimal sampling periods for various MCU and control methods. All figures are given for the case of active communication with the design computer via the serial interface so that monitoring and data acquisition are still possible. According to our experience, while a sampling time of 5 ms allows stable levitation and reference tracking, control performance visibly improves with shorter sampling times. The CircuitPython implementation is a special case because unpredictable memory operations cannot guarantee strictly real-time operation.

The following discussion will not repeat a thorough mathematical description of well-known control engineering methods. However, in an educational setting, tutorials tailored to the worked examples may be of value to students.

#### 4.2.1. PID Control

Perhaps all introductory control engineering and mechatronics courses culminate with lectures on the omnipresent PID control method. Worked examples for PID are implemented in C/C++, CircuitPython, Simulink and REXYGEN and for various standard prototyping boards.

**Figure 21:**
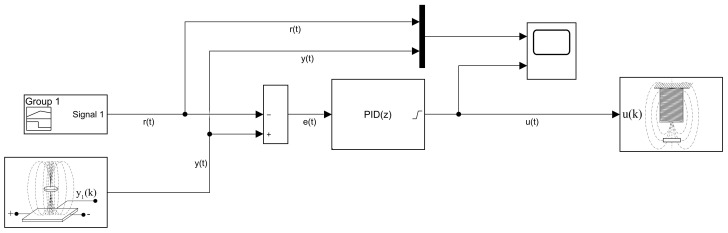
Simulink scheme for PID control of magnetic levitation using MagnetoShield.

An example of PID trajectory tracking implemented using the C/C++ API is shown in Figure 20. The pre-set reference levels are tracked accurately, albeit with noticeable transients that exhibit a significant overshoot effect, illustrating this specific control phenomenon for educational purposes. It is important to note that the PID controller used in this demonstration was empirically tuned to achieve these results. While implementing the PID algorithm from scratch is an excellent learning exercise, the AutomationShield library also provides ready-to-use PID control classes. Integrating a functional control loop for the MagnetoShield can be as straightforward as employing the pre-built PID block from the Simulink library and integrating it with the input–output blocks of the MagnetoShield Simulink API, as depicted in Figure 21.

#### 4.2.2. State Estimation

Prior to continuation with model-based control algorithms, it is necessary to employ means of acquiring all the internal states of the system. Naturally, the position of the permanent magnet is measured and the current can be obtained via measurements likewise, but furthermore, we need the velocity of the magnet. Since the system is observable, we may use some type of estimation in this case. The easiest way is, of course, the numerical differentiation of the position; unfortunately, in the worked examples below, this method was considered quite unreliable due to the noise sensitivity. Examples for educational purposes with Luenberger observer were introduced (git) but the examples focusing on particular control algorithms usually use Kalman filtering. Kalman filtration is also beneficial for making the measurements of the current more precise.

#### 4.2.3. Pole Placement and Linear Quadratic Control

**Figure 22:**
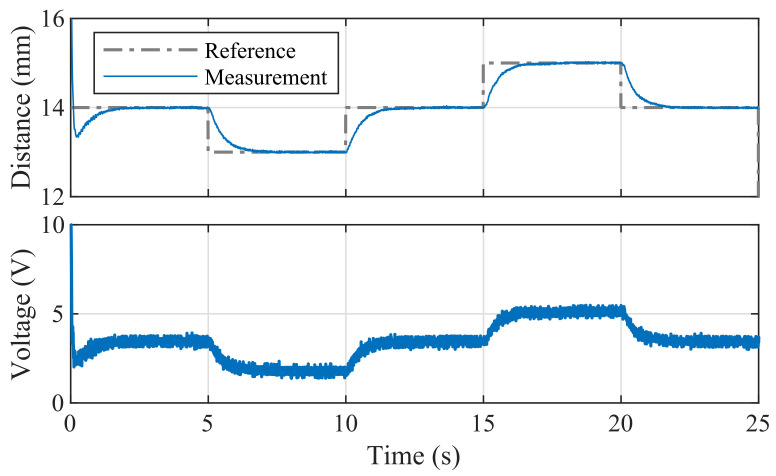
Experiment with pole-placement-based control implemented using Arduino IDE API.

Figure 22 illustrates an example of pole-placement-based control implemented in C/C++ using Arduino IDE API, which can be also found in the library. In this demonstration, stabilizing poles were chosen so as to provide an effective control of the magnetic levitation process.

**Figure 23:**
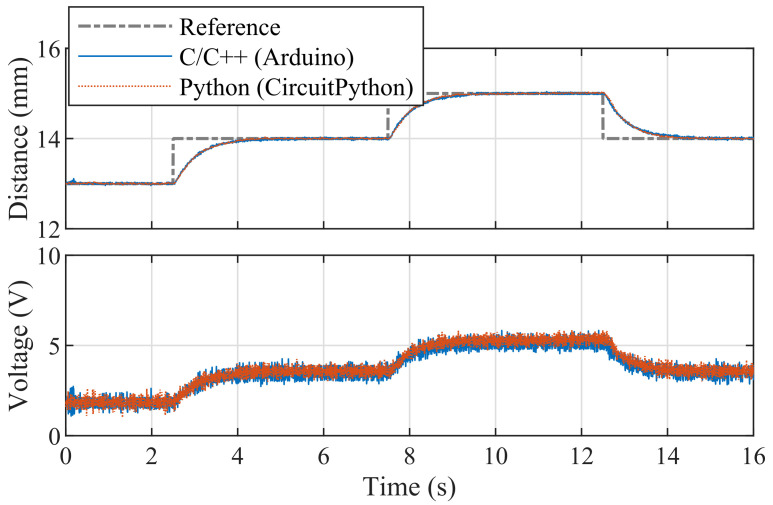
Experiment with LQ control implemented using Arduino IDE and Python APIs.

Figure 23 illustrates linear quadratic control of the levitation height for the C/C++ implementation in Arduino IDE and the pseudo-real-time version using CircuitPython. Despite CircuitPython being implemented in non-strict real-time, there is no appreciable difference in the tracking quality. As expected, the responses differ from PID control (see Figure 20) mainly in the transients, where LQ removes the overshoot effect.

#### 4.2.4. Model Predictive Control

Finally, let us inspect whether microcontroller hardware with limited computational power may be used to teach and research the principles of optimal control, specifically the constrained linear model predictive control (MPC) [48,49]. Worked examples for MPC on the MagnetoShield provided with the AutomationShield library make use of two distinct development lines of the same control concept: processor-intensive implicit optimization performed online and memory-critical explicit MPC (EMPC). Creating a custom solver is not a trivial task; therefore, we have resorted to the μAO-MPC [50] package for implicit MPC and the Multi-Parametric Toolbox [51] for explicit MPC.

In order to minimize the computational burden, MPC examples are formulated with input-only constraints (0–10 V) and do not contain a stabilizing terminal set.

The low-end ATmega328p microcontroller found in the ubiquitous Arduino Uno represents the imaginary border of hardware capability when it comes to linear MPC. Although both implicit MPC [52] and explicit MPC [53] have been successfully deployed on this MCU in real time before, these have been either slower and simpler models or highly modified versions of the conventional algorithms. The more complex model and higher sampling time of magnetic levitation are prohibitive for the Uno from the viewpoint of a volatile memory footprint. Examples for both variations in MPC are provided for the Arduino IDE (MagnetoShield_MPC.ino, MagnetoShield_EMPC.ino) and Simulink. A prediction horizon of seven steps is attainable on the Arduino Mega2560 with 5 ms sampling considering the sub-optimal first-order solver of μAO-MPC, while the non-volatile memory of the MCU is nearly filled with the lookup tables from the explicit MPC problem with merely four steps. Higher class MCUs allow considerably more leeway in the problem formulation.

**Figure 24:**
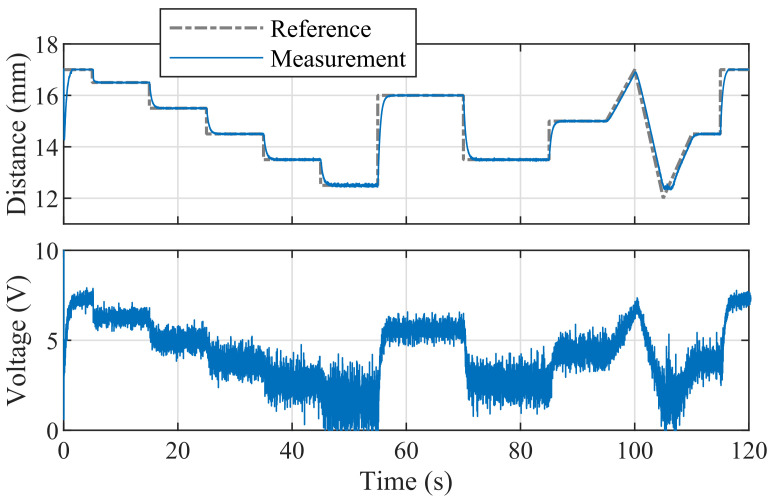
Experiment with explicit MPC implemented on Arduino Due using Simulink API.

Worked examples of the model predictive control of magnetic levitation are provided for the Arduino mega2560 and Due boards. Figure 24 demonstrates the EMPC tracking of a reference distance implemented in Simulink and deployed on the Arduino Due. The controller performance gained by the more advanced control method is somewhat diminished by the longer sampling period.

As has been outlined previously, even methods of lesser complexity such as LQ are only borderline implementable on current hardware running a CircuitPython interpreter. Therefore, we have not attempted to port implicit MPC solvers for the MagnetoShield. An explicit MPC solution has been tested on the ATSAMD51 since the memory capability of hardware is adequate. Nevertheless, an online direct sequential search algorithm results in sampling times well over 5 ms—rendering levitation unattainable due to the fast dynamics of the process.

### 4.3. Experience from the Educational Process

The MagnetoShield, besides other devices developed under the AutomationShield project, has been integrated into our Microprocessor Technology undergraduate course as a part of the educational process. Students in this course learn the fundamentals of microcontroller application development and programming skills through hands-on experience. At the end of the semester, they are required to write a C++ library and develop custom classes that will create an API for the MagnetoShield. The API should be capable of measuring calibrated sensor readings and driving the actuator with simple commands. The MagnetoShield is also being utilized in our graduate courses on the System Identification Toolbox as a demonstration of various concepts such as

- Basic simplifications;
- RLC analogies;
- Closed-loop identification;
- Frequency domain identification.

In the Theory of Automatic Control course, the MagnetoShield is used as a classroom demonstration before being handed out to students for take-home experiments to design and fine-tune model-based control algorithms, such as LQ and MPC.

In the previous semester, the MagnetoShield was introduced as a benchmark design for data acquisition and analysis in the Measurement of Technical Quantities course. The use of hardware in these courses has received positive feedback from students, despite encountering some minor implementation issues. Some students found the noisy sensor data and the hands-on approach to tuning the calculated controllers for better performance to be particularly beneficial. They claimed that personal experience with the differences between nominal systems and controllers and their practical realizations was highly educational. However, we are continuously working to eliminate workarounds and complete error management to ensure that the devices are as plug-and-play as possible. We also plan to develop tutorials and study materials to make it easier for students to focus on the core of these courses.

## 5. Conclusions

In this article, we have presented a compact and affordable open-source magnetic levitation device that can be used for both education and research purposes. The detailed hardware design and mathematical model derivation, system identification, and validation provide a solid foundation for anyone who wishes to reproduce or build upon our work. We still recognize room for improvement regarding either the hardware or software; since the AutomationShield initiative is driven by the open-source philosophy, anyone is welcome to contribute and collaborate.

The easy-to-use open-source API in C/C++ for the Arduino IDE, Simulink, and CircuitPython enables users to customize the control algorithms and test different scenarios with ease. Our calculations demonstrate that the material cost of this device can be less than USD 34, making it accessible to individuals and institutions with limited budgets.

Looking ahead, we plan to extensively use and examine the capabilities of this device as an educational tool in control engineering courses. We envision that this device can be a valuable platform for hands-on experimentation and project-based learning, enabling students to better understand and apply concepts in control theory. Furthermore, we anticipate exploring the implementation and testing of additional control algorithms beyond the already implemented PID, LQ, and MPC. In summary, this work provides a comprehensive and accessible approach to magnetic levitation experimentation that can be used by anyone interested in learning about control engineering or exploring new control algorithms. The affordability and ease of use make it a valuable tool for students, educators, and researchers alike.

## Figures and Tables

**Table 1:** Bill of materials and price list in U.S. dollars for a single device or quantities near ∼100 pieces sorted based on descending price. The small quantity figure has been optimized for ease of accessibility, while the large quantity for price using a meta-search engine. The includes only direct material price and excludes postage, equipment, consumables, energy and labor cost.

Name	Part Number, Value	PCB		Pcs.	Unit	Total	Unit	Total
					Large quantity		Small quantity	
Current sensor	Texas Instruments INA169 (e.g., TI INA169NA/250)	U4	(h)	1	1.57	1.57	3.47	3.47
Trimmer	10 kΩ, 250 mW, multi turn, SMD, 10% (e.g., Bourns 3214W-1-103E)	R10	(l)	1	0.74	0.74	3.81	3.81
Hall sensor	Texas Instruments DRV5055Z4 (e.g., DRV5055Z4QDBZR)	U2	(c)	1	0.95	0.95	1.69	1.69
Electromagnet	2.5 kg suction, ϕ 20 × 15 mm, 24 V (e.g., Bairun Electric BR-20/15)	L1	(b)	1	0.91	0.91	5.53	5.53
Opamp	dual, rail-to-rail (e.g., Texas Instruments TLC2272ACD)	U5	(f)	1	0.69	0.69	2.18	2.18
DAC	Microchip MCP4725 (e.g., MCP4725A0T-E/CH)	U3	(e)	1	0.38	0.38	1.15	1.15
PCB	2-layer, FR4, 1.6 mm thick (e.g., JCLPCB)	-	(x)	1	0.40	0.40	2.91	2.91
3D print	11 g, ϕ = 1.75 mm PETG filament, bright green, at 240 °C (90 °C bed), 2 h	-	(y_1_)–(y_5_)	1	0.39	0.39	2.36	2.36
Trimmer	10 kΩ, 250 mW, single turn, (e.g., ACP CA14NV12,5-10KA2020)	POT1	(t)	1	0.19	0.19	0.20	0.20
Screw	nylon, M5 × 0.8, Phillips Pan, machine screw (e.g., Essentra 50M050080P010)	-	(B)	1	0.16	0.16	0.22	0.22
Resistor	3 kΩ, 0805, 0.1%, 0.125 W (e.g., Viking AR05BTCW3001)	R6, R9	(q),(r)	2	0.16	0.31	0.48	0.96
Diode	generic, DO214AC (e.g., Vishay Semiconductor BYG20J)	D1	(n)	1	0.15	0.15	0.44	0.44
Screw	nylon, M4 × 0.7, Phillips Pan, machine screw (e.g., Essentra 50M040070D006)	-	(A)	1	0.12	0.12	0.19	0.19
Header	10 × 1, female, stackable, 0.1″ pitch (e.g., SparkFun 474-PRT-10007)	-	(i_3_)	1	0.09	0.09	0.38	0.38
Magnet	NdFeB, disc, ϕ = 9 mm, *h* = 2 mm, N50 (e.g., OMO Magnets N50D00960020)	-	(a)	1	0.09	0.09	0.15	0.15
Resistor	1 kΩ, 0805, 0.1%, 0.125 W (e.g., Viking AR05BTCW1001)	R7	(s)	1	0.08	0.08	0.23	0.23
Resistor	10 Ω, 0805, 0.1%, 0.1 W (e.g., ROYAL OHM TC0525B0100T5)	R8	(p)	1	0.08	0.08	0.48	0.48
Transistor	BJT transistor, NPN, SOT-23-3 (e.g., Nexperia BC817K-40HVL)	Q1	(g)	1	0.08	0.08	0.30	0.30
Pot shaft	For “POT1”, (e.g., ACP CA9MA9005)	-	(u)	1	0.07	0.07	0.14	0.14
Header	8 × 1, female, stackable, 0.1″ pitch (e.g., SparkFun 474-PRT-10007)	-	(i_2_)	2	0.06	0.12	0.38	0.76
Header	6 × 1, female, stackable, 0.1″ pitch (e.g., SparkFun 474-PRT-10007)	-	(i_1_)	1	0.06	0.06	0.38	0.38
Resistor	4.7 kΩ, 0805, 0.5%, 0.125 W (e.g., Panasonic ERA-6AED472V)	R3	(m)	1	0.05	0.05	0.29	0.29
LED	0805, red (e.g., OPTOSUPPLY OSR50805C1E)	D2	(v)	1	0.04	0.04	0.22	0.22
Tube	acryl (PMMA), *h* = 10 mm, ϕ = 12/10 mm (e.g., Bitspower BP-NCCLT12AC)	-	(z)	1	0.03	0.03	0.51	0.51
Zener diode	3.3 V, SOD323 (e.g., NEXPERIA BZX384-C3V3.115)	D3–D5	(o_1_)–(o_3_)	3	0.03	0.08	0.05	0.15
Capacitor	0805, ceramic, 0.1 μF (e.g., KEMET C0805C104K3RACTU)	C1, C3	(k_1_), (k_2_)	2	0.02	0.05	0.30	0.60
Header	1 × 2 female, 0.1″ pitch (e.g., Connfly DS1023-1*2S21)	-	(i_5_)	1	0.02	0.02	0.11	0.11
Resistor	1 kΩ, 0805, 5%, 0.125 W (e.g., Royal Ohm 0805S8J0102T5E)	R5	(w)	1	0.01	0.01	0.12	0.12
Resistor	10 kΩ, 0805, 5%, 0.125 W (e.g., ROHM Semiconductor SFR10EZPJ103)	R1, R2	(j_1_), (j_2_)	2	0.01	0.02	0.14	0.29
Header	1 × 2 male, 0.1″ pitch (e.g., Amphenol G800W306018EU)	-	(i_4_)	1	0.01	0.01	0.12	0.12
Glue	Single component epoxy (e.g., Suxun B-7000)	-	-	0.1 mL	0.01	0.01	0.01	0.01
Postage	-	-	-	-	-	-	-	3.23
					Total:	$7.96		$33.57

**Table 2:** Execution time for the basic serial communication-based single-input single-output operation of the MagnetoShield using various software.

	Execution Time (ms)		
**Software**	**Average**	**Std. Dev.**	**Maximum**
MATLAB 2021b	64	14	344
MATLAB 2021b (Arduino Due)	78	14	314
Scilab 6.1.0	20	6	106
LabView 2020	24	1	25
Octave 6.1.0	44	4	66

**Table 3:** Initial and identified model parameters. (†—measured, ‡—for the mechanical and electrical equation).

Parameter	Initial	Identified	Unit
*L*	0.235 ^†^	0.391	H
*R*	210 ^†^	214	Ω
*K*	$1.5\;\times \;10^{-9}$	$6.0\;\times \;10^{-9}/4.4\;\times \;10^{-7}$ ^‡^	N · A^−1^
*m*	$0.74\;\times \;10^{-3}$ ^†^	-	kg
*x*_01_	0.015 ^†^	-	m
*x*_02_	0.0	-	m · s^−1^
*x*_03_	0.024 ^†^	-	A
*u*_0_	5.00 ^†^	-	V

**Table 4:** Demonstration examples for various closed-loop control techniques implemented for different API. ^†^—pseudo real-time, but levitation is attained. ^‡^—not possible to implement because of performance issues. N/A—untested.

	PID	PP	LQ	MPC	EMPC
C/C++ (Arduino)	✓	✓	✓	✓	✓
Python (CircuitPython)	✓ ^†^	✓ ^†^	✓ ^†^	✗ ^‡^	✗ ^‡^
Simulink	✓	✓	✓	✓	✓
REXYGEN	✓	N/A	N/A	N/A	N/A

**Table 5:** Illustration of the range of feedback control examples on various hardware. Limitations ^‡^—memory. ${\;}^{★}$ —untested but hardware implementability assumed to be attainable using at least one API.

MCU (Prototype Board)	Clock (MHz)	ROM (kB)	RAM (kB)	PID	PP	LQ	MPC	EMPC
ATmega328p (Arduino Uno)	16	32	2	✓	✓	✓	✗ ^‡^	✗ ^‡^
ATmega2560 (Arduino Mega)	16	256	8	✓	✓	✓	✓	✓
SAM3X8E (Arduino Due)	84	512	96	✓	✓	✓	✓	✓
ATSAMD21G18 (Adafruit Metro M0 Express)	48	256	32	✓ ${\;}^{★}$	✓ ${\;}^{★}$	✓ ${\;}^{★}$	✓ ${\;}^{★}$	✓ ${\;}^{★}$
ATSAMD51 (Adafruit Metro M4 Express)	120	512	192	✓ ${\;}^{★}$	✓ ${\;}^{★}$	✓ ${\;}^{★}$	✓ ${\;}^{★}$	✓ ${\;}^{★}$

**Table 6:** Minimum recommended sampling time in ms for various closed-loop control strategies.

MCU	PID	PP	LQ	MPC	EMPC
ATmega328p (Arduino Uno)	2.0	2.5	2.5	-	-
ATmega2560 (Arduino Mega)	2.0	2.5	2.5	5.0	5.0
SAM3X8E (Arduino Due)	1.0	1.5	1.5	3.0	3.0

## Data Availability

The data presented in this study are available on request from the corresponding author. The data are not publicly available due to privacy.
